# Structural Characterization and Electrochemical Studies of Selected Alkaloid N-Oxides

**DOI:** 10.3390/molecules29122721

**Published:** 2024-06-07

**Authors:** Olha Dushna, Liliya Dubenska, Andrzej Gawor, Jakub Karasińki, Oksana Barabash, Yurii Ostapiuk, Mykola Blazheyevskiy, Ewa Bulska

**Affiliations:** 1 Biological and Chemical Research Centre, Faculty of Chemistry, University of Warsaw, Żwirki i Wigury 101, 02-093 Warsaw, Poland; o.dushna@cnbc.uw.edu.pl (O.D.); agawor@chem.uw.edu.pl (A.G.); jkarasinski@chem.uw.edu.pl (J.K.); 2 Faculty of Chemistry, Ivan Franko National University of Lviv, Kyryla i Mefodiya 6, 79-005 Lviv, Ukraine; liliya.dubenska@lnu.edu.ua (L.D.); oksana.barabash@lnu.edu.ua (O.B.); yurii.ostapiuk@lnu.edu.ua (Y.O.); 3 Department of General Chemistry, National University of Pharmacy, Valentynivska 4, 61-168 Kharkiv, Ukraine; blazejowski@ukr.net

**Keywords:** alkaloids, N-oxide alkaloids, synthesis, mass spectrometry, FT-IR spectroscopy

## Abstract

In this work, we synthesized and confirmed the structure of several alkaloid N-oxides using mass spectrometry and Fourier-transform infrared spectroscopy. We also investigated their reduction mechanisms using voltammetry. For the first time, we obtained alkaloid N-oxides using an oxidation reaction with potassium peroxymonosulfate as an oxidant. The structure was established based on the obtained fragmentation mass spectra recorded by LC-Q-ToF-MS. In the FT-IR spectra of the alkaloid N-oxides, characteristic signals of N-O group vibrations were recorded (bands in the range of 928 cm⁻^1^ to 971 cm⁻^1^), confirming the presence of this functional group. Electrochemical reduction studies demonstrated the reduction of alkaloid N-oxides at mercury-based electrodes back to the original form of the alkaloid. For the first time, the products of the electrochemical reduction of alkaloid N-oxides were detected by mass spectrometry. The findings provide insights into the structural characteristics and reduction behaviors of alkaloid N-oxides, offering implications for pharmacological and biochemical applications. This research contributes to a better understanding of alkaloid metabolism and degradation processes, with potential implications for drug development and environmental science.

## 1. Introduction

Alkaloids (ALs) constitute a large group of substances of plant and synthetic origin that are extensively utilized by humans [1]. Alkaloids have a variety of biological activities and are widely used in medicine [2,3,4]. The ALs are utilized in the formulation of anti-inflammatory, analgesic, stimulating, antimicrobial, anticancer, antifungal, antispasmodic, neuropharmacological, and other drugs [5,6,7]. Recent studies [8,9] have also shown the potential usefulness of some ALs in the fight against SARS-CoV-2, specifically berberine, an alkaloid found in the fruits of barberry and turmeric, and quinine, an alkaloid from the quinoline series. These ALs have been demonstrated to have the ability to inhibit viral replication, suggesting potential applications in the treatment of SARS-CoV-2 [8,9].

In addition, plants containing ALs are a common component of the human diet, present in both food and beverages [10]. Some well-known examples of ALs found in the human diet include caffeine from coffee seeds, theobromine from cocoa seeds, theophylline from tea leaves, tomatine from tomatoes, and solanine from potatoes [2].

Some of the ALs, including those widely used in pharmacy, have a toxic effect on humans and animals. The misuse of ALs such as morphine, cocaine, caffeine, and nicotine can have fatal consequences [11].

Consuming food products that contain alkaloids can also cause disease [12]. Several pyrrolizidine and tropane ALs, widely distributed in plants and plant products, have been identified as hepatotoxic and carcinogenic, representing one of the most dangerous classes of phytotoxins affecting the peripheral and central nervous systems, especially the organs involved in digestion and metabolism [13,14,15]. In accordance with European recommendations (Commission Regulation (EU) 2021/1399 [16], Commission Regulation (EU) 2020/2040 [17], Commission Regulation (EU) 2016/239 [18]), control limits for tropane alkaloids (atropine, scopolamine) are set at 1 μg/kg in plant-origin products, while pyrrolizidine alkaloids range from 1 μg/kg to 750 μg/kg, depending on the matrix. For example, in tea, the AL content should not exceed 1 μg/kg, and in herbal infusions of lemon balm and peppermint, the limit is set at 400 μg/kg. Therefore, the careful control of food products and the regulation of AL use in medicine are important current tasks.

ALs undergo various biochemical transformations in living organisms. Major metabolic pathways, facilitated by hepatic enzymes, encompass hydrolysis, conjugation, and oxidation [19]. AL N-oxides are typical intermediates of AL metabolism and are often excreted from the body in this form. However, oxides can also exhibit bioactivity; in particular, N-oxides of the pyrrolizidine alkaloids, the formed oxidation products, can readily react with proteins, create DNA adducts, and induce tumors via a genotoxic mechanism [20,21]. On the other hand, some alkaloid N-oxides formed during the N-oxidation reaction are considered safe markers with no harmful effects on the human body and are easily excreted through urine [22]. For instance, the primary metabolites of the nicotine alkaloid are cotinine and nicotine N-oxide. An average smoker, consuming 10 cigarettes daily, excretes approximately 0.56 mg of nicotine N-oxide unchanged in the urine within 24 h [23,24,25]. This indicates that alkaloid N-oxides formed during the metabolic process could potentially function as markers for monitoring alkaloids in biological fluids, especially concerning the consumption of alkaloid-based medications and food items containing alkaloids.

Moreover, alkaloid N-oxides represent a common form of existence for many alkaloids in plants [26]. Therefore, it is crucial to monitor the content of food products and plant materials such as tea and honey, emphasizing not only the alkaloids themselves but also their N-oxides. For the identification and determination of the alkaloid N-oxides in various objects, there is a need for their standard solutions. Therefore, a standardized methodology for the synthesis of alkaloid N-oxides is required.

This investigation aimed to establish an environmentally sustainable and economically viable methodology for the synthesis of alkaloid N-oxides while validating their structural integrity. This endeavor involved the utilization of mass spectrometry and infrared spectroscopy. In contemporary laboratory settings, the integration of high-performance chromatography with mass spectrometry detection (MS or MS/MS) is prevalent for the analysis of various substances, including food products and herbal medicinal raw materials. Therefore, augmenting the existing database and discerning the disparities between the mass spectra of alkaloids and their N-oxide counterparts are imperative tasks. The study of infrared spectra of alkaloid N-oxides opens the possibility of rapid and cheap determination of such compounds.

The subsequent phase of our research focused on elucidating the mechanism underlying the reduction process involved in synthesizing alkaloid N-oxides, which may mimic metabolic or degradation reactions occurring in vivo. This investigation holds particular significance given the potential physiological relevance of such reactions. In recent years, a combined approach leveraging electrochemical (EC) methods alongside mass spectrometry (MS) has emerged as a powerful tool for addressing this challenge [27,28]. By coupling EC methods with MS detection, this approach not only complements existing biochemical studies techniques but also offers advantages in terms of efficiency and cost-effectiveness. Moreover, it facilitates the identification of electrochemical reaction products, shedding light on their potential roles as metabolic or degradation by-products. This integrated methodology represents a cornerstone in the fields of metabolomics and bioanalytical chemistry, providing valuable insights into complex biological processes and chemical transformations [28,29,30,31,32].

**Figure 1:**
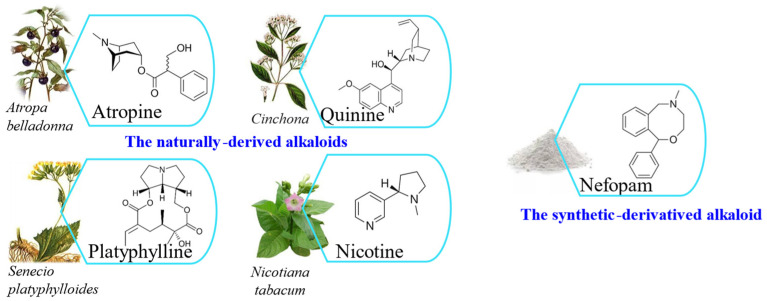
The alkaloids employed in this study were utilized for the synthesis of alkaloid N-oxides.

This study focused on five alkaloids that belong to different chemical groups and serve different purposes. These include both natural and synthetic compounds, as depicted in Figure 1. Specifically, the alkaloids selected are atropine (a tropane), quinine (a quinuclidine), platyphylline (a pyrrolizidine), nicotine (a pyridine), and nefopam (a synthetic benzoxazocine). This selection is strategically diverse, enabling a comprehensive examination of various classes of alkaloids, each renowned for its unique pharmacological activities and widespread utility in medical applications. It should be added that alkaloid N-oxides are formed regardless of the type of alkaloid skeleton. The process typically involves the oxidation of a nitrogen atom in the alkaloid structure to form an N-oxide functional group. This transformation can occur during an oxidation reaction. The presence of N-oxide groups can affect the pharmacological properties of the alkaloid, including its bioavailability, metabolic stability, and interaction with biological targets. Thus, investigating the potential formation of alkaloid N-oxides with different skeletal types can be a valuable aspect of research, providing insight into their metabolic pathways.

## 2. Results and Discussion

### 2.1. Synthesis of Alkaloid N-Oxides

Alkaloid N-oxides can be easily synthesized in the laboratory [33]. Hydrogen peroxide and organic peroxyacids are commonly employed as oxidants [34,35,36,37,38]. The commercial reagent potassium peroxymonosulfate (KPMS) was used as an oxidant, enabling the synthesis of the main metabolites of alkaloids in just 15 min [39,40,41,42]. The oxidation of alkaloids is rapid at room temperature, except for nicotine. The formation of nicotine N-oxide at 20–25 °C takes 40 min, and slight heating of the solution accelerates the reaction. A 5-fold excess of KPMS should also be used.

The main factor affecting the completeness of the oxidation of an alkaloid to its N-oxide is pH. Alkaloids are oxidized to their N-oxides in an alkaline environment. The optimum pH for oxidation is close to the pKa of the alkaloid. Table 1 shows the optimal conditions for obtaining the studied alkaloid N-oxides. Previously, we reported the preparation of nefopam N-oxide [39], nicotine N-oxide [42], platyphylline N-oxide [40], and atropine N-oxide [41]. In this study, we present, for the first time, the synthesis of quinine N-oxide via this specific method and oxidation reaction (Table 1). Thus, we have standardized the methodology for the synthesis of alkaloid N-oxides.

**Scheme 1:**
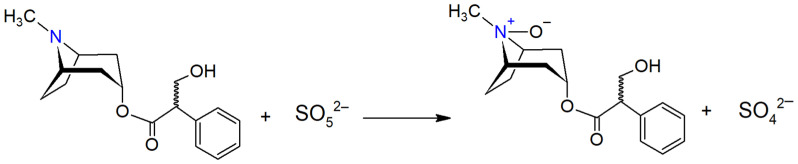
The reaction of obtaining atropine N-oxide using the oxidant peroxymonosulfate.

The oxidation reaction of atropine with peroxymonosulfate to atropine N-oxide is depicted in Scheme 1. This second-order reaction has a rate constant of 0.193 L·mol^−1^·min^−1^, and its kinetics adhere to the principles of specific acid-base catalysis [41].

### 2.2. Studies on Structure of Alkaloid N-Oxides by Mass Spectrometry

Identification and characterization of alkaloid N-oxides were achieved by liquid chromatography coupled with high-resolution mass spectrometry (LC-Q-ToF-MS). Mass spectra were recorded for both the alkaloids and the synthetically obtained alkaloid N-oxides to identify the obtained N-oxides and confirm the presence of an additional oxygen atom in the structure. Table 2 presents the measured and theoretically calculated values of [M + H]^+^ for all the studied compounds. Moreover, the mass accuracy was calculated to confirm the chemical formula of the investigated compounds. The mass accuracy for compounds was computed by calculating the absolute difference between the theoretical and experimentally observed mass-to-charge ratios (*m*/*z*), dividing this difference by the theoretical *m*/*z*, and expressing the result in parts per million (ppm). The obtained values of mass accuracy did not exceed the permissible threshold of 5 ppm, which is deemed acceptable for high-resolution mass spectrometry [43,44] and reliably confirms that N-oxides ALs were obtained.

**Figure 2:**
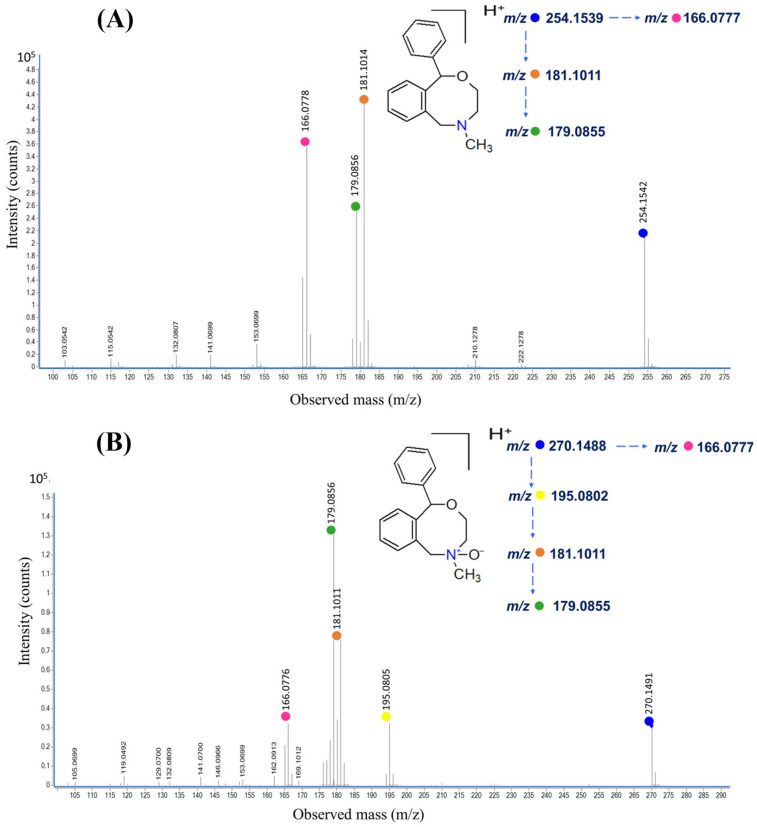
Elucidation of fragmentation patterns of nefopam (**A**) and nefopam N-oxide (**B**).

**Figure 3:**
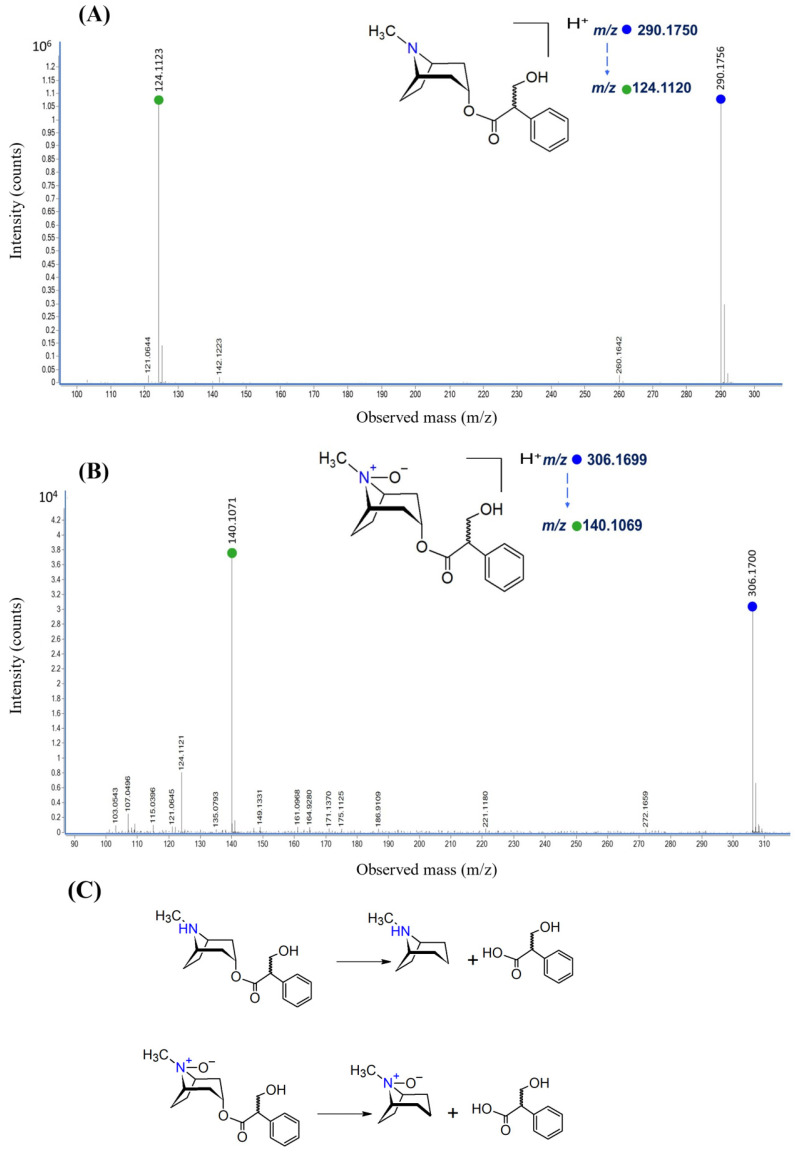
Elucidation of fragmentation patterns of atropine (**A**) and atropine N-oxide (**B**) and proposed fragmentation pattern of atropine and atropine N-oxide (**C**).

**Figure 4:**
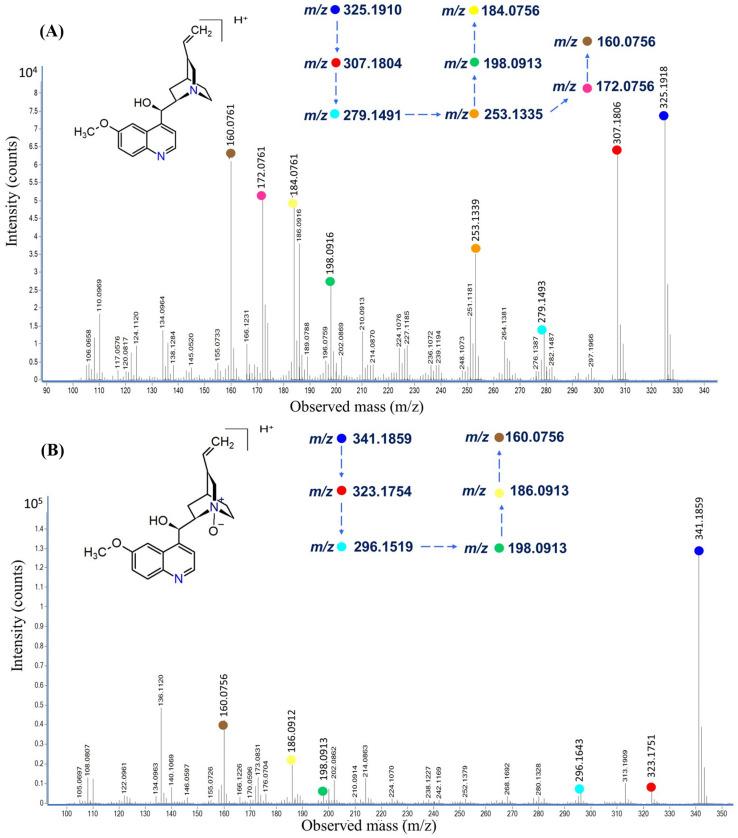
Elucidation of fragmentation patterns of quinine (**A**) and quinine N-oxide (**B**).

**Figure 5:**
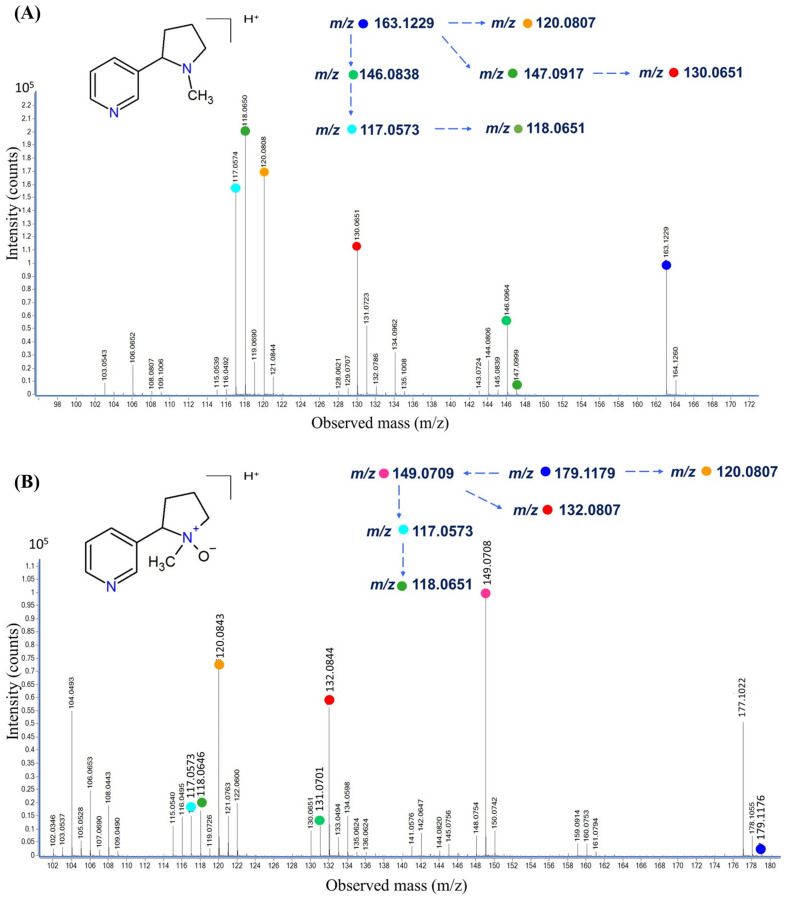
Elucidation of fragmentation patterns of nicotine (**A**) and nicotine N-oxide (**B**).

An essential prerequisite for the structure elucidation of alkaloid N-oxides was a comprehensive understanding of the fragmentation process of these compounds. Below is a detailed interpretation of the mass spectra of selected alkaloid N-oxides acquired in MS^2^. The following pairs of compounds were studied as research compounds: quinine and its N-oxide, atropine and its N-oxide, nefopam and its N-oxide, and nicotine and its N-oxide. Figure 2, Figure 3, Figure 4 and Figure 5 illustrate the fragmentation mass spectra of study compounds where the *m*/*z* values correspond to the exact mass values on the mass spectrum. The obtained *m*/*z* values of the fragmented ions were compared with the theoretical values. Figure 2, Figure 3, Figure 4 and Figure 5 show theoretical values for the proposed schemes. Figure 3C and Figures S1–S3 (Supplementary Materials) show the detailed fragmentation scheme of alkaloids and alkaloid N-oxides.

#### 2.2.1. Nefopam and Nefopam N-Oxide

In the fragmentation mass spectrum of the protonated molecular ion of nefopam at *m*/*z* 254, product ions at *m*/*z* 181, 179, and 166 were observed, as obtained in Figure 2A. The major fission fragment product ion at *m*/*z* 181 resulted from a loss of C_3_H_7_NO moiety from the benzoxazocine ring. Further detachment of two hydrogen atoms from an ion at *m*/*z* 181 will lead to the formation of an ion at *m*/*z* 179. Accurate mass measurement of these ions confirmed their chemical formulas. The fragmentation pathways are given in Figure S3 (Supplementary Materials). The obtained mass spectrum is fully consistent with previous research provided by Yu et al. [45].

In the fragmentation mass spectrum of nefopam N-oxide, a pattern like that of the fragmentation mass spectrum of nefopam was observed. However, a small fraction of nefopam N-oxide underwent fragmentation, resulting in the formation of an ion at *m*/*z* 195. This suggests the cleavage of the C_3_H_9_NO fragment from the protonated molecular ion of nefopam N-oxide (*m*/*z* 270) (Figure 2B). These results align with those described by Yu et al. [45].

#### 2.2.2. Atropine and Atropine N-Oxide

The fragmentation of the protonated molecular ion of atropine results in the main product ion at *m*/*z* 124 (C_8_H_13_N) and of atropine N-oxide at *m*/*z* 140 (C_8_H_13_NO) (Figure 3). Additionally, a signal at *m*/*z* 142 was detected on the fragmentation mass spectrum for atropine, serving as a secondary product of atropine fragmentation. These results align with those described by Chen et al. and Luo et al. [46,47].

#### 2.2.3. Quinine and Quinine N-Oxide

On the fragmentation mass spectrum of the protonated molecular ion of quinine at *m*/*z* 325, product ions at *m*/*z* 307, 279, 253, 198, 186, 184, 172, and 160 were observed (Figure 4A). Also, we registered for the protonated molecular ion of quinine N-oxide at *m*/*z* 341 and product ions at *m*/*z* 323, 296, 198, 186, and 160 (Figure 4B). The obtained fragmentation mass spectrum of quinine is in complete agreement with prior studies [48,49]. Furthermore, we compared the two obtained mass spectra of quinine and quinine N-oxide among ourselves. The signals at *m*/*z* 186 and 160 correspond to the fragmentation of the quinoline ring, and these signals are present in both mass spectra. The difference between the signals at *m*/*z* 307 for quinine and *m*/*z* 323 for quinine N-oxide is 16, indicating that the oxidation of quinine occurs at the tertiary nitrogen atom in the quinuclidine fragment.

#### 2.2.4. Nicotine and Nicotine N-Oxide

The fragmentation mass spectrum of the protonated molecular ion of nicotine at *m*/*z* 163 observed product ions at *m*/*z* 146, 147, 130, 120, 118, and 117 (Figure 5A). Similarly, for the protonated molecular ion of nicotine N-oxide at *m*/*z* 179, we registered product ions at *m*/*z* 179, 149, 132, 120, 118, and 117 (Figure 5B). We also compared the two mass spectra of nicotine and nicotine N-oxide. The signals present in both mass spectra at *m*/*z* 120, 118, and 117 correspond to protonated fragments of pyridine derivatives. This is indicative of the formation of nicotine N-oxide by the piperidine fragment. These results agree with Liang et al., Smyth et al., and Tsugawa et al., as described by [50,51,52,53]. However, we believe that the fragmentation of nicotine and nicotine N-oxide is more likely to occur, as depicted in Figure S2 (Supplementary Materials).

### 2.3. Studies on Structure of N-Oxide Alkaloids by FT-IR Spectrometry

**Figure 6:**
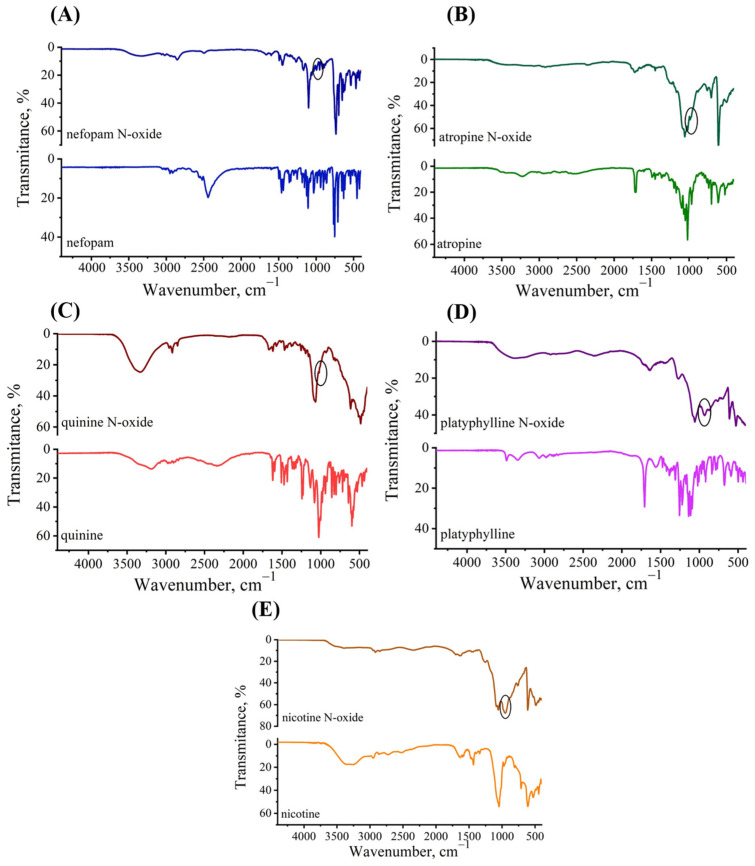
The FT-IR spectra of alkaloid N-oxides and alkaloids: (**A**) nefopam N-oxide and nefopam; (**B**) atropine N-oxide and atropine; (**C**) quinine N-oxide and quinine; (**D**) platyphylline N-oxide and platyphylline; (**E**) nicotine N-oxide and nicotine. The N-O group vibration signal is highlighted in circles.

The IR spectra of alkaloids and their alkaloid N-oxides, presented in Figure 6, indicate specific characteristics of the molecular structure of the investigated compounds. Table 3 provides vibration signals for all groups present in the structures of alkaloids and their N-oxides. From the IR spectra data, a notable distinction between the spectra of alkaloids and their N-oxides is the presence of N-O group vibrations (bands in the range of 928 cm⁻^1^ to 971 cm⁻^1^). These bands, characteristic of the N-O stretch, are crucial for identifying N-oxides but are relatively weak and fall into the fingerprint region, making them less prominent. The N-O group vibration signal is highlighted in Figure 6.

The region from 2500 cm⁻^1^ to 3660 cm⁻^1^ is represented by broad peaks corresponding to the stretching of O–H and C–H bonds, indicating the presence of active hydrogen bonds in the molecules of alkaloid N-oxides.

Additionally, these peaks also suggest the presence of hydroxyl and methyl groups. Peaks in the range of 1403 cm⁻^1^ to 1608 cm⁻^1^ reflect vibrations of aromatic systems (C=C and C–C), indicating the existence of aromatic ring systems in the molecules of alkaloids, particularly in nicotine, quinine, and atropine. Furthermore, in the case of platyphylline and atropine N-oxides, absorption bands in the range of 1451 cm⁻^1^ to 1637 cm⁻^1^ were identified, corresponding to the vibrations of C=O in the alkaloid molecules, suggesting the presence of carbonyl group in the structure of these molecules. In general, the rest of the IR spectroscopic data of investigated alkaloid N-oxides agree with those reported in the literature [49,54,55,56].

The N–O group vibration signal is highlighted in a black circle.

### 2.4. Study of Alkaloid N-Oxide Reduction with the Electrochemical Approach

The electrochemical (EC) approach was employed to gain deeper insights into the metabolism and degradation mechanisms of alkaloid N-oxides in biological systems. Nowak et al. extensively reviewed the simulation of drug metabolism through various methods, including EC [57]. From a pharmaceutical perspective, the EC approach offers several advantages, such as being cost-effective, easy to use, and avoiding ethical concerns associated with using human or animal materials. This makes it a highly appealing option for drug metabolism research [58]. This approach was also used in this work.

**Figure 7:**
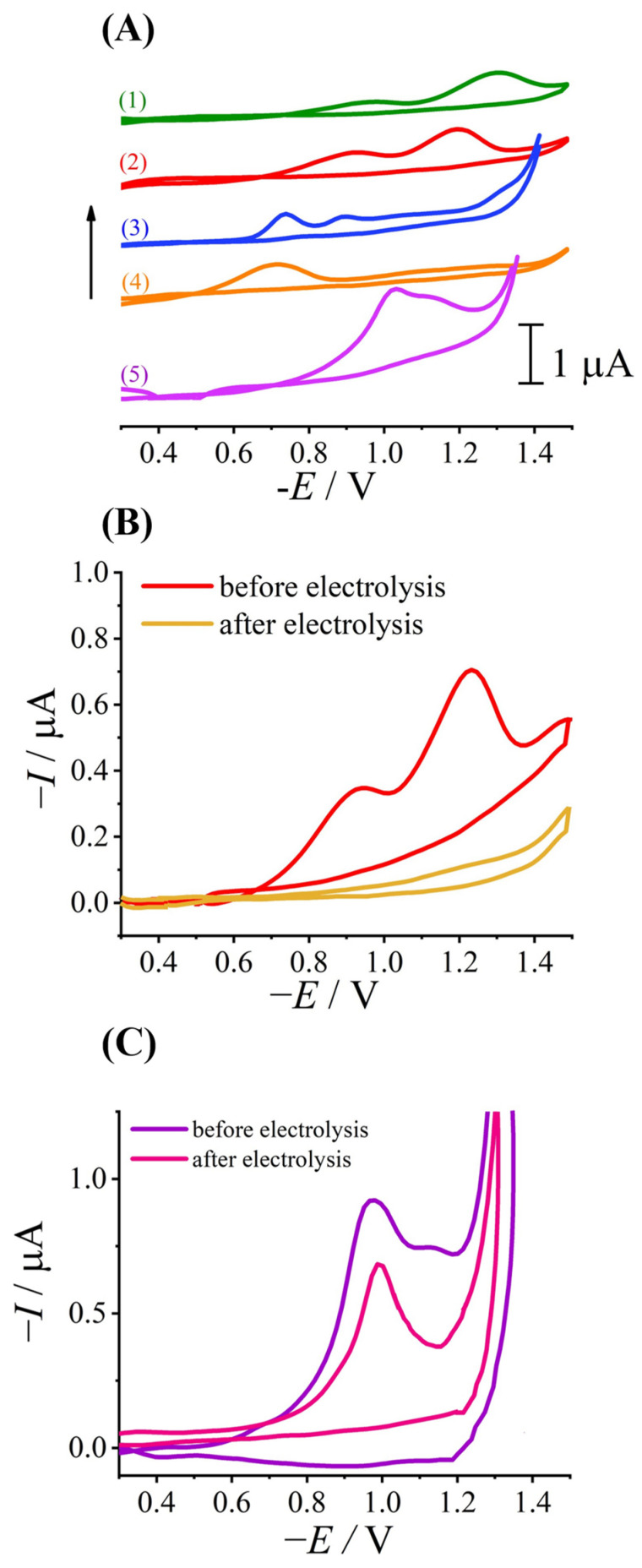
(**A**) Cyclic polarograms of atropine N-oxide (1), nefopam N-oxide (2), nicotine N-oxide (3), platyphylline N-oxide (4), and quinine N-oxide (5) under optimal conditions. The concentration of alkaloid N-oxides was 40 μmol/L. Scan rate—0.5 V/s. (**B**) Cyclic polarograms of nefopam N-oxide before and after electrolysis. The concentration of nefopam N-oxide was 20 μmol/L. Scan rate—0.5 V/s. (**C**) Cyclic polarograms of quinine N-oxide before and after electrolysis. The concentration of quinine N-oxide was 20 μmol/L. Scan rate—0.5 V/s.

Alkaloid N-oxides are easily reduced on the surface of mercury-based electrodes over a wide pH range, resulting in the formation of either one or two peaks (depending on the alkaloid N-oxides and the pH of the solution). Figure 7A shows the polarograms of the reduction of alkaloid N-oxides obtained in optimal conditions [39,40,41,42]. The arrow in Figure 7A indicates that polarograms of reduced alkaloid N-oxides were shifted up towards axis *Y* for a better view. Previously, we selected optimal conditions for the reduction of alkaloid N-oxides, including the pH of reduction and the scan rates. In this work, for the first time, we investigated the electrochemical behavior of quinine N-oxide; namely, the optimal conditions for its reduction on the surface of a dropping mercury electrode were established. On the dropping mercury electrode (DME), quinine N-oxide is reduced in the form of a single peak at a potential of −1.15 V (Figure 7A). It should be noted that the first peak on the polarogram of quinine N-oxide (E = −1.03 V—Figure 7A) corresponds to the reduction of quinine itself, according to [59].

It was determined that the reduction current of all research alkaloid N-oxides exhibits a diffusion–adsorption nature. The optimal environment for the reduction of alkaloid N-oxides is within the pH range of 3 to 7, which is provided by the Britton–Robinson buffer (BRB).

Also, we investigated and described the mechanism of alkaloid N-oxide reduction on the surface of mercury electrodes [39,40,41,42]. When we obtained the two peaks of the reduction of the N-oxides of the alkaloids, this process corresponds to two single-electron transfer processes. The first single-electron transfer to the N-oxide yields an amine radical cation together with a hydroxide ion, and the second reduction yields the original structure of the alkaloid. This process is reflected in the appearance of two peaks on the voltammogram, as observed in the reduction of atropine N-oxide and nefopam N-oxide [39,41]. In the case of the polarogram of alkaloid N-oxides showing a single cathodic peak, it indicates the one-stage reduction of the alkaloid N-oxide, involving one electron and one proton. In this context, the reduction of the alkaloid N-oxide yields a hydroxylamine derivative. Subsequently, this hydroxylamine derivative can revert to the original alkaloid through a protonation reaction in a mildly acidic environment. However, this process cannot be registered under voltammetry conditions. This one-step reduction process of alkaloid N-oxides has been observed for nicotine N-oxide and platyphylline N-oxide [40,42]. It is important to note that both proposed mechanisms of reduction of alkaloid N-oxides are agreed on by data in the literature [60,61].

To confirm the hypothesis regarding the mechanism of alkaloid N-oxide reduction on the electrode surface, identification and structural determination were carried out using mass spectrometry (MS). Initially, prolonged electrolysis of solutions of alkaloid N-oxides was performed using a macro electrode of mercury as the working electrode (to increase the surface area and accelerate the process). Electrolysis was conducted for two compounds at a concentration of 20 µM for 6 h: the nefopam N-oxide at the potential of the first peak (*E* = −1.00 V) and the quinine N-oxide at the potential of the peak (*E* = −1.15 V). By maintaining such experimental conditions, we aimed to ensure that the reduction reaction selectively targeted the alkaloid N-oxide compound in accordance with the specified potential. The primary objective of the prolonged electrolysis duration was to facilitate the generation of the principal reduction product of the alkaloid N-oxide on the surface of the mercury electrodes. Throughout this process, we meticulously tracked and observed the emergence of only one product, aligning precisely with the compound described within the theoretical framework of the reduction mechanism. The polarograms of the reduction nefopam N-oxide and quinine N-oxide before and after electrolysis are shown in Figure 7B,C. Other research shows that alkaloids do not reduce at the DME.

During electrolysis, the signals of reduction alkaloid N-oxides decreased, and after 6 h, they completely disappeared. This indicates the complete reduction of alkaloid N-oxides. The same pattern was observed in the chromatograms obtained by the LC-Q-ToF-MS method. The reduction peak at *E* = −1.03 V (Figure 7C) was registered on the polarogram of quinine after 6 h of electrolysis, which corresponds to the reduction of quinine itself and not its N-oxide (see above).

The mass spectra for nefopam N-oxide and its electrolysis product were received. We have established that the nefopam N-oxide ([M + H]^+^ = 270.1480) is reduced to the nefopam ([M + H]^+^ = 254.5434). Similarly, the quinine N-oxide is reduced to the protonated anionnitroxide radical derivative ([M + H]^+^ = 342.1929), which, in turn, transforms back to the quinine ([M + H]^+^ = 325.1864) due to protonation.

Thus, we have confirmed the reduction of alkaloid N-oxides on the electrode surface to the original alkaloids. This detailed mass spectrometric analysis provides clear evidence for the reduction mechanisms proposed. Furthermore, the identification of intermediate products and final reduction compounds through MS offers a deeper understanding of the electrochemical reduction pathways and confirms the stepwise reduction processes.

This electrochemical approach offers advantages in terms of ease of handling, practicality, and environmental friendliness, presenting a viable alternative to conventional oxidation or reduction reactions commonly employed in organic biomolecule synthesis. Given the benefits of electrochemical synthesis in producing target molecules, it holds promising prospects for surpassing limitations associated with traditional synthesis methods. Moreover, the ability to simulate metabolic pathways and degradation processes in a controlled and ethical manner enhances the relevance of this approach in pharmaceutical and biochemical research, providing novel insights into the behavior of alkaloid N-oxides and their potential applications in drug development and analysis.

## 3. Materials and Methods

### 3.1. Chemical and Reagents

Following alkaloid substances were used in this work: nefopam hydrochloride, atropine sulfate, nicotine sulfate, platyphylline hydrotartrate, and quinine hydrochloride. Detailed characteristics of alkaloid substances are given in Table 4. Potassium peroxymonosulfate (KPMS) (CAS No. 70693-62-8) was used as an oxidizing agent. The Britton–Robinson buffer (BRB) was used as a background electrolyte. To prepare the BRB, 20.2 g of Na_2_B_4_O_7_·10H_2_O, 28.7 mL of CH_3_COOH, and 17.6 mL of H_3_PO_4_ were dissolved in the volumetric flask [39,40,41,42]. Analytical reagent-grade chemicals were purchased from Sigma-Aldrich (Darmstadt, Germany), EMD Millipore (Darmstadt, Germany), and Baker (Deventer, The Netherlands). Samples were diluted with deionized water obtained by the Milli-Q System (resistivity 18.2 MΩ cm; EMD Millipore, Darmstadt, Germany). Formic acid (~98%) used for liquid chromatographic purposes was purchased from Honeywell Chemicals (Bracknell, UK).

### 3.2. Instrumentation

#### 3.2.1. Mass Spectrometry

An Agilent 1290 Infinity system (Agilent Technologies, Wood Dale, IL, USA) coupled with an Agilent 6450 UHD Accurate-Mass Q-TOF mass spectrometer (Agilent Technologies, Wood Dale, IL, USA) was used to obtain high-resolution mass spectra. Electrospray ionization (ESI) in positive ion mode was applied. ESI conditions were as follows: nebulizing gas temperature, 300 °C; nebulizing gas flow, 7 L/min; sheath gas temperature, 350 °C; and sheath gas flow, 11 L/min. Nitrogen was used as a nebulizer and sheath gas. The collision energy was 20 eV. Full-scan spectrums and fragmentation mass spectrums were acquired in the (100–1000) *m*/*z* range.

The chromatographic separation of the compounds was carried out using a Synergi 2.5 μm Polar-RP 100 Å, 100 mm × 2 mm column, and gradient elution. Phase A (0.1% formic acid in water) and phase B (0.1% formic acid in acetonitrile) were used as mobile phases. The injection volume was 2 µL. Mass spectra were obtained for the studied compounds at a concentration of approximately 5 μmol/L in a solution of 1:1 methanol/water.

#### 3.2.2. FT-IR Spectroscopy

The samples of alkaloids and alkaloid N-oxides in dry form were analyzed with a Fourier-transform infrared spectrophotometer IRSpirit FTIR (Shimadzu, Japan), equipped with a diamond-tipped ATR accessory. The data were collected in absorbance mode, and the wavelength ranged from 4400 cm^−1^ to 400 cm^−1^. A spectral resolution of 4 cm^−1^ was used. Software LabSolutions IR 2.13 (Shimadzu, Japan) was used for spectrum collection and spectrum processing.

To transfer to a dry form, we extracted the alkaloid N-oxides in dry form. Specifically, alkaloid N-oxides were extracted from the aqueous solution using 10 mL of chloroform in three separate extractions. Subsequently, the extracts containing alkaloid N-oxides in chloroform were dried at room temperature until dry crystals formed.

#### 3.2.3. Electrochemistry

We used the digital device MTech OVA-410 (MTech Lab, Lviv, Ukraine) with three-electrode cell (working dropping mercury electrode (DME), a saturated calomel reference electrode, and platinum wire auxiliary electrode) [62]. The applied DME had *τ* = 12 s in 0.1 mol/L NH_4_Cl with an open circuit. The current was measured at a fixed time (10 s) in the life of the drop.

Cyclic voltammetry and linear sweep voltammetry with a scan rate of 0.5 V/s were used to study the mechanism of reduction of alkaloid N-oxides. The prepared solutions for measurement were transferred to an electrochemical cell, and dissolved oxygen was removed with purified argon for 10 min. Polarograms were recorded in the potential range from 0 V to −1.5 V with a scan rate of 0.5 V/s (and back for cyclic voltammetry).

To establish the detailed structure of the products of the electrochemical reaction of the reduction of alkaloid N-oxides, a long electrolysis was carried out for 6 h using mercury macroelectrode as a working electrode.

## 4. Conclusions

In this work, for the first time, we comprehensively investigated some synthesized alkaloid N-oxides using KPMS as an oxidant. The standardized methodology developed here for the synthesis of alkaloid N-oxides is promising for pharmaceutical research, facilitating their production for further study and offering avenues for new drug development.

Mass spectrometry and Fourier-transform infrared spectroscopy were used to establish and confirm the structure. Using electrochemical methods, in particular voltammetry, the mechanism of reduction of alkaloid N-oxides on the surface of mercury electrodes was confirmed. For the first time, we have isolated the products of electrochemical reduction of alkaloid N-oxides and determined their structures using high-resolution mass spectrometry. It turned out that most alkaloid N-oxides are restored to the original form of the alkaloid itself. Such elucidation of reduction mechanisms using electrochemical methods in combination with mass spectrometry is a significant step forward in revealing alkaloid metabolism and degradation pathways. Understanding the electrochemical behavior of alkaloid N-oxides on electrode surfaces sheds light on potential reactions in living organisms. This research is critical to understanding the fate and effects of alkaloids and has implications for drug metabolism and toxicity studies.

In addition, the obtained results improve our ability to monitor the content of alkaloids and their N-oxides in food products, thereby improving food safety practices. Overall, this comprehensive study represents a significant advance in alkaloid N-oxide research spanning pharmacology, toxicology, and food science. Further research in this area holds great potential for harnessing the benefits of alkaloids and their derivatives in biomedical and agricultural contexts.

## Figures and Tables

**Table 1:** Optimal conditions for obtaining alkaloids N-oxides.

	Alkaloids	Nefopam N-Oxide [39]	Nicotine N-Oxide [42]	Platyphylline N-Oxide [40]	Atropine N-Oxide [41]	Quinine N-Oxide
Optimal Conditions						
pH_oxidation_ (provided by BRB)		8.0	9.3	8.4	10.2	9.5
Oxidation temperature, °C		20–25	40	20–25	20–25	20–25

**Table 2:** Characteristics of the mass spectra of the studied compounds.

Compound	Chemical Formula	Theoretical [M + H]^+^	Measured [M + H]^+^	Mass Accuracy, ppm
Nefopam	C_17_H_19_NO	254.1539	254.1542	1.18
Nefopam N-oxide	C_17_H_19_NO_2_	270.1488	270.1491	1.11
Atropine	C_17_H_23_NO_3_	290.1750	290.1756	2.07
Atropine N-oxide	C_17_H_23_NO_4_	306.1699	306.1700	0.33
Quinine	C_20_H_24_N_2_O_2_	325.1910	325.1918	2.46
Quinine N-oxide	C_20_H_24_N_2_O_3_	341.1859	341.1859	0
Platyphylline	C_18_H_27_NO_5_	338.1969	338.1964	−1.48
Platyphylline N-oxide	C_18_H_27_NO_6_	354.1918	354.1927	2.54
Nicotine	C_10_H_14_N_2_	163.1229	163.1229	0
Nicotine N-oxide	C_10_H_14_N_2_O	179.1179	179.1176	−1.67

**Table 3:** Selected assigned IR vibrations of alkaloids and their N-oxides.

Alkaloids	Experimental Wavenumbers, cm^−1^ (Signal Intensity) ^a^		Type of Vibration and Bond	Functional Group
	Alkaloids	Alkaloids N-Oxide		
Atropine	3500–3223 w	3660–2500 w	ν ^b^ (O–H)	Hydroxy
	3080 w	3066 w	ν (C–H)	Phenyl
	1721 m	1724 m	ν (C=O)	Carboxylic
	1494 w	1403–1500 w	ν (C=C)	Phenyl
	1200 m	1229 m	ν (C–N)	Tropane
	–		ν (N–O)	N-oxide group
	–	971 s		
Nefopam	3044 w	3063 w	ν (C–H)	Phenyl
	1600 w	1600 w	ν (C–C)	Phenyl
	1497–1437 m	1446–1493 m	ν (C=C)	Phenyl
	1240 m	1267 m	ν (C–N)	Benzoxazocine
	–	952 m	ν (N–O)	N-oxide
Quinine	3550–3053 m	3600–3000 m	ν (O–H)	Hydroxy
	2943 w	2955 w	ν (C–H)	Quinoline
	1618 m	1617 m	ν (C–C)	Quinoline
	1647–1594 m	1668–1571 w	ν (C=N)	Quinoline
	1473–1431 m	1467–1431 m	ν (C=C)	Quinoline
	1235 w	1253 m	ν (C–N)	Quinuclidine
	1025 s	1066 s	ν (C–O)	6-Methoxyquinoline
	–	928 s	ν (N–O)	N-oxide group
Platyffiline	3488–3210 w	3488–3200 w	ν (O–H)	Hydroxy
	3067 w	2937 w	ν (C–H)	12-Hydroxysenecionan
	2977 w	2919 w	ν (C–H)	12-Hydroxysenecionan
	1707 m	1705 w	ν (C=O)	12-Hydroxysenecionan-11,16-dione
	1554 w	1552 w	ν (C–C)	12-Hydroxysenecionan-11,16-dione
	1437 w	1437 w	ν (C–O)	12-Hydroxysenecionan-11,16-dione
	1241 m	1270 m	ν (C–N)	Pyrrolizidine
	–	931 s	ν (N–O)	N-oxide
Nicotine	3360–3079 m	3250–3000 m	ν (C–H)	Pyridine
	2950 w	2952 w	ν (C–H)	1-Methylpyrrolidin
	1628 m	1628 m	ν (C–C)	Pyridine
	1643–1530 w	1650–1579 m	ν (C=N)	Pyridine
	1473–1433 m	1467–1431 m	ν (C=C)	Pyridine
	1220 w	1253 m	ν (C–N)	Pyrrolidine
	–	948 s	ν (N–O)	N-oxide

^a^ Intensity: s = strong; m = medium; w = weak. ^b^ Symbol ν means stretching deformation.

**Table 4:** Characteristics of alkaloid substances.

Compound	CAS No.	Molecular Mass	pKa	Purity, %
Nefopam	23327-57-3	253.34	8.98	≥99.0
Nicotine	54-11-5	162.23	8.02	99.0
Atropine	5908-99-6	289.36	9.84	99.7
Platyphylline	480-78-4	337.41	8.1	98.5
Quinine	6119-47-7	324.18	8.0	≥99.0

## Data Availability

Data are contained within the article and Supplementary Materials.

## Supplementary Materials

The following supporting information can be downloaded at https://www.mdpi.com/article/10.3390/molecules29122721/s1. Figure S1. Proposed fragmentation pattern of quinine (A) and quinine N-oxide (B). Figure S2. Proposed fragmentation pattern of nicotine (A) and nicotine N-oxide (B). Figure S3. Proposed fragmentation pattern of nefopam (A) and nefopam N-oxide (B).
