# Supplementary material for: Structural Characterization and Electrochemical Studies of Selected Alkaloid N-Oxides
Source: Molecules. 2024 Jun 7;29(12):2721. doi: 10.3390/molecules29122721 (PMC11205554; doi:10.3390/molecules29122721)
Supplement: Supplementary file 1 [file molecules-29-02721-s001.zip › molecules-3034520-supplementary.pdf]

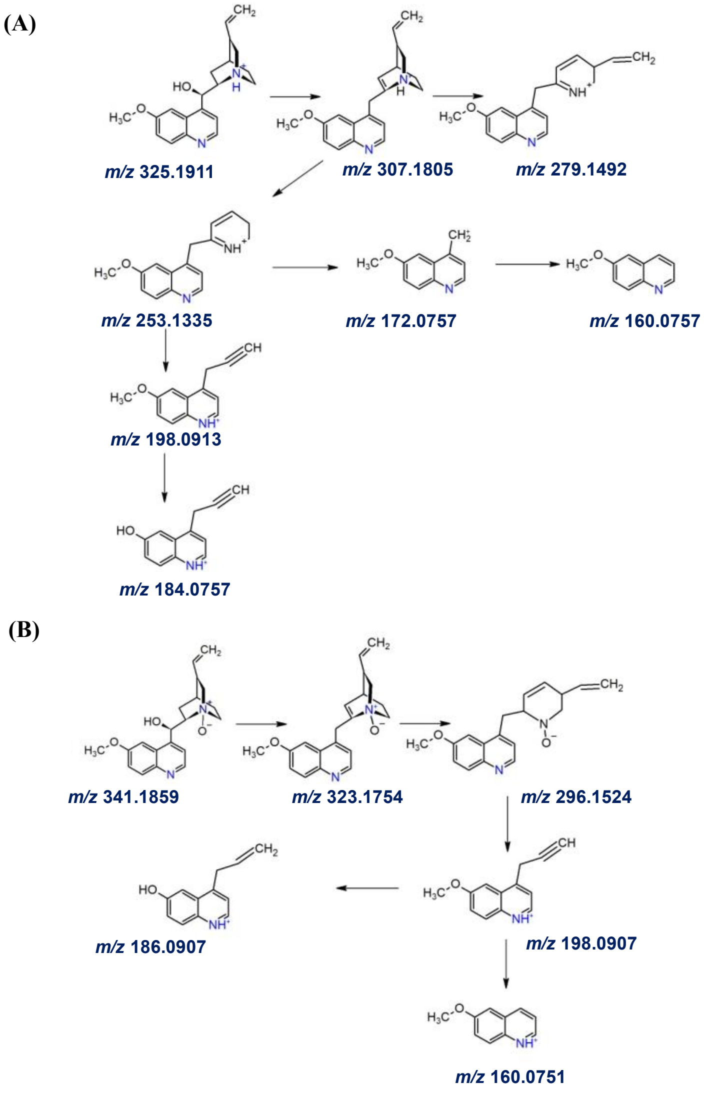

Figure S1. Proposed fragmentation pattern of quinine (A) and quinine N-oxide (B).

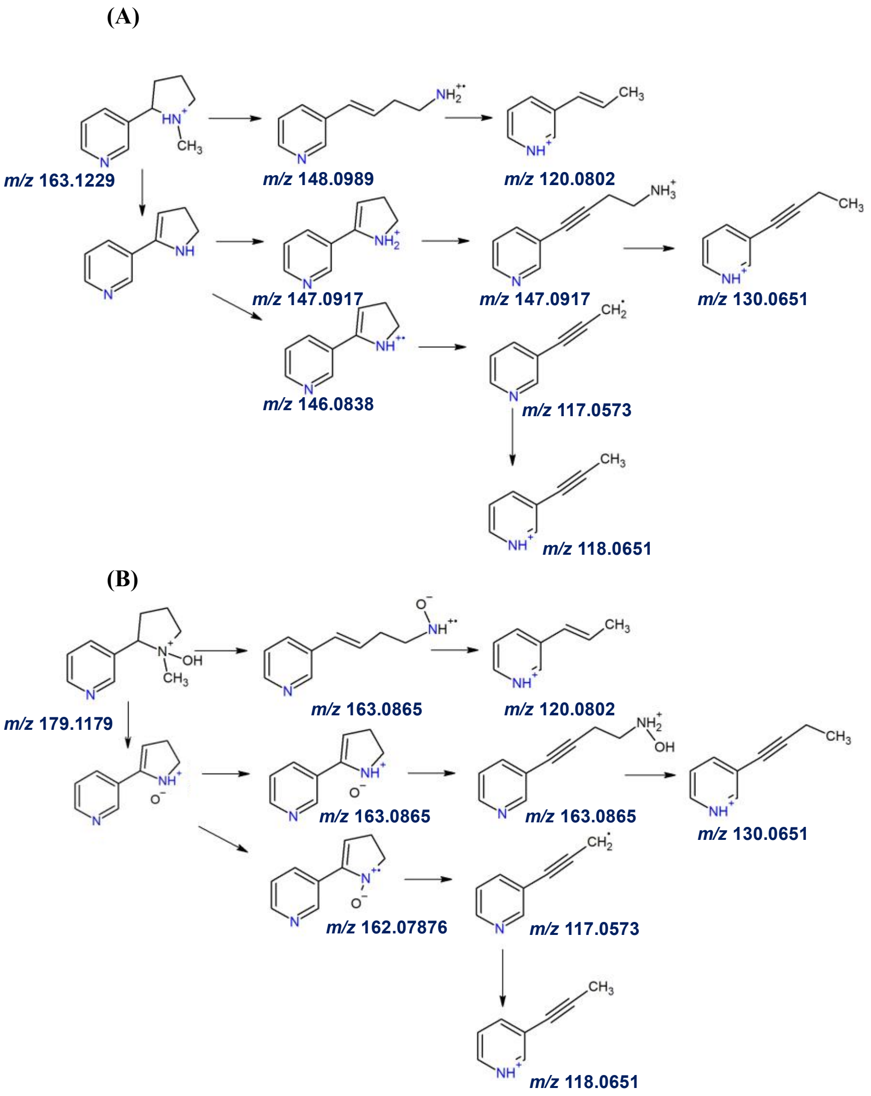

Figure S2. Proposed fragmentation pattern of nicotine (A) and nicotine N-oxide (B).

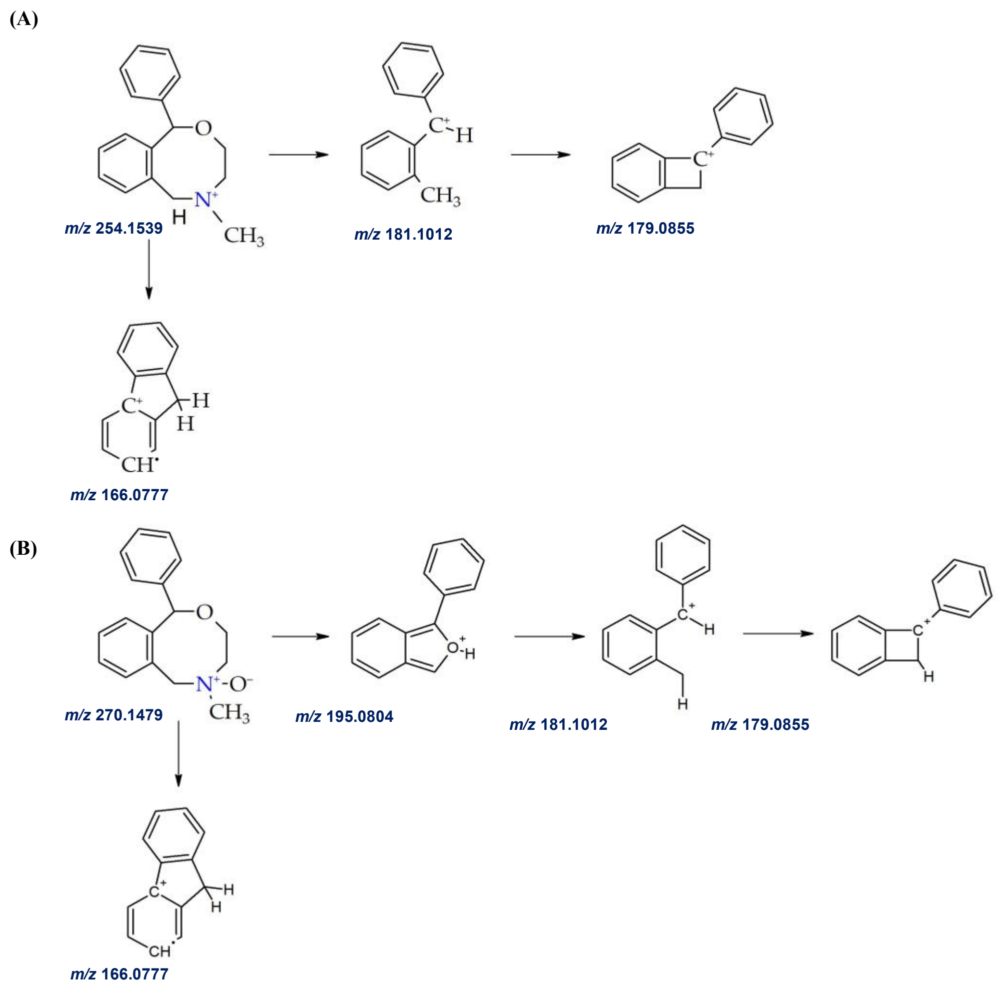

Figure S3. Proposed fragmentation pattern of nefopam (A) and nefopam N-oxide (B).
